# Olaparib and advanced ovarian cancer: Summary of the past and looking into the future

**DOI:** 10.3389/fphar.2023.1162665

**Published:** 2023-04-21

**Authors:** Brigida Anna Maiorano, Mauro Francesco Pio Maiorano, Evaristo Maiello

**Affiliations:** ^1^ Oncology Unit, Foundation Casa Sollievo della Sofferenza IRCCS, San Giovanni Rotondo, Italy; ^2^ Department of Translational Medicine and Surgery, Catholic University of the Sacred Heart, Rome, Italy; ^3^ Division of Obstetrics and Gynecology, Biomedical and Human Oncological Science, University of Bari “Aldo Moro”, Bari, Italy

**Keywords:** olaparib (Lynparza^TM^), PARP, ovarian cancer, BRCA, target therapy

## Abstract

Ovarian cancer (OC) is women’s eighth most common cancer, bearing the highest mortality rates of all female reproductive system malignancies. Poly (ADP-ribose) polymerase inhibitors (PARPis) have reshaped the treatment scenario of metastatic OC as a maintenance post platinum-based chemotherapy. Olaparib is the first PARPi developed for this disease. Results from Study 42, Study 19, SOLO2, OPINION, SOLO1, and PAOLA-1 clinical trials, led to the FDA and EMA approval of olaparib for the maintenance treatment of women with high-grade epithelial ovarian, fallopian tube, or primary peritoneal cancer without platinum progression: in the platinum-sensitive recurrent OC; in the newly diagnosed setting in case Breast Cancer (BRCA) mutations and, in combination with bevacizumab, in case of BRCA mutation or deficiency of homologous recombination genes. In this review, we synthetized olaparib’s pharmacokinetic and pharmacodynamic properties and its use in special populations. We summarized the efficacy and safety of the studies leading to the current approvals and discussed the future developments of this agent.

## 1 Introduction

With an incidence of 8.1 cases/100,000 inhabitants/year, ovarian cancer (OC) is the eighth most common cancer among women worldwide. It accounts for more deaths than any other malignancy of the female reproductive system, bearing a mortality rate of 5.4 deaths/100,000 inhabitants/year. Most OC cases are diagnosed as metastatic (57%), with a 5-year survival rate of only 30.8% (Siegel et al., 2022; Cancer stat facts: ovarian cancer, 2023). Platinum-based chemotherapy (CHT) represents the first choice in the metastatic setting of OC. However, despite initial benefits, over 2 out of 3 patients will relapse within the first 2 years (McGuire et al., 1996; Neijt et al., 2000; Piccart et al., 2000; Ozols et al., 2003; Armstrong et al., 2006; Kehoe et al., 2015; Walker et al., 2019). Poly-(ADP-ribose)-polymerase (PARP) inhibitors (PARPis) are a class of antitumor agents whose mechanism of action relies on the exploitation of the defective DNA repair pathways in Breast Cancer (BRCA) mutant and Homologous Recombination (HR) repair genes deficient (HRD) cells, a group of crucial genes for double-stranded breaks (DSBs) and interstrand crosslinks (ICLs) repairing pathways, a process notably known as “synthetic lethality” (Farmer et al., 2005; Lord and Ashworth, 2012). Of note, half of all OCs are associated with HRD, and 22% of cases bear a germline or somatic mutation of BRCA1 and BRCA2, thus indicating the use of PARPis as a possible target therapy for OC (The Cancer Genome Atlas Research Network, 2011). Olaparib (LYNPARZA^®^, AstraZeneca Pharmaceuticals LP), a potent inhibitor of human PARP-1, PARP-2, and PARP-3, is historically the first PARPi developed and approved for the clinical use of metastatic OC. Currently, olaparib is approved in USA and EU for the maintenance treatment of women with high-grade (HG) epithelial ovarian, fallopian tube, or primary peritoneal cancer, if BRCA1/2-mutated (germline or somatic) in the first line, or platinum-sensitive relapsed OC (PS-ROC), after any response (complete or partial) to platinum-based CHT. In combination with bevacizumab, olaparib is approved in case of HRD after any response to platinum-based CHT (FDA approved Olaparib, 2019; EMA Olaparib product information, 2023).

In our review, we aimed to summarize the pharmacological properties, therapeutic efficacy, and tolerability of olaparib, examining its role and use in treating advanced OC.

## 2 Pharmacodynamic properties of olaparib

*In vitro*, olaparib inhibits PARP-1, -2, and -3 with IC50 5, 1, and 4 nM, respectively. It also has weak activity against PARP-5a (tankyrase 1 [TNKS1]) with IC50 1,500 nM (Committee for Medicinal Products for Human, 2014; US Food and Drug Administration FDA, 2014; McCormick et al., 2018; Zhou et al., 2019) (Table 1).

**TABLE 1:** Pharmacokinetics and pharmacodynamics of olaparib.

	Dose (mg)	Cmax (ng/mL)	Tmax (h)	T1/2 (h)	IC50 (nM)	Metabolism	Cytocrome metabolism
Olaparib	300/12 h	7,700	1.5	11.9	PARP1: 5, PARP2: 1, PARP3: 4, PARP5a: 1500	Liver (42% recovered in feces), kidney (44% recovered in urine)	CYP 3A4/5 with 3 metabolites: M12 (ring opened hydroxy-cyclopropyl), M15 (mono-oxygenated), M18 (dehydrogenated piperazine)
	400/12 h	9,300	2				

CYP3A4/5, cytochrome P 3A4/5; PARP1/2/3, Poly (ADP-ribose) polymerase 1/2/3.

Similarly to other PARPis, olaparib acts through the mechanism of “synthetic lethality,” as it inhibits PARP enzymes, causing the accumulation of DNA damage. In the case of HRD, this inhibition leads to apoptosis. Moreover, olaparib causes cytotoxic and pro-apoptotic PARP-DNA trapping. In pre-clinical models, these effects seemed additive or synergistic with the cytotoxicity exerted on DNA by chemotherapeutic agents, with even more contribution to DNA fragmentation and cell apoptosis than olaparib alone (McCormick et al., 2018). Among resistance mechanisms, BRCA reversion mutations that restore the HR function are the main findings in olaparib-resistant cells. Moreover, the occurrence of somatic mutations which restore the open reading frame of HRR genes, defects in non-homologous end-joining, increased drug efflux [e.g., with mutations of P-glycoprotein (P-gp)], or loss of 53BP1, have been found (Noordermeer and van Attikum, 2019).

## 3 Pharmacokinetic properties of olaparib

At the daily dosage of 600 mg tablets divided into two administrations (BID), olaparib’s mean maximum plasma concentration (Cmax) is 7,700 ng/mL, reached in a median time (Tmax) of 1.5 h, and the half-life is 14.9 h. Olaparib is available as capsules or tablets. The two formulations are not equivalent: as evidenced by different studies, the 300 mg tablets had a 13% higher mean relative exposure at the steady state than the 400 mg capsules. In the case of 400 mg BID, Cmax is around 9,300 ng/mL, and Tmax is around 2 h (Dean et al., 2012; Yamamoto et al., 2012; Mateo et al., 2016; Yonemori et al., 2016; Plummer et al., 2018; Yuan et al., 2019) (Table 1). Cytochromes P450 (CYP)3A4 and -5 mainly metabolize olaparib, forming three principal metabolites: M12 (ring opened hydroxy-cyclopropyl) M15 (mono-oxygenated), and M18 (dehydrogenated piperazine), with the potency to inhibit the growth of BRCA1-mutant cells and PARP-1 30-fold, 30-fold and 4-fold lower than olaparib, respectively (Committee for Medicinal Products for Human, 2014). The use of potent inhibitors of CYP3A, such as clarithromycin, erythromycin, diltiazem, itraconazole, ketoconazole, ritonavir, verapamil, goldenseal, and grapefruit, increases the Cmax of olaparib of 42% [90% confidence interval (CI), 33%–52%] and the median area under the curve (AUC) of 170% (90% CI, 144%–197%). Thus, co-administration is not recommended unless the dose of olaparib is reduced to 100 mg or 150 mg BID if a potent or moderate inhibitor is used, respectively. Olaparib also weakly inhibits CYP3A4 *in vitro* and CYP3A *in vivo*, thus possibly increasing the exposure to CYP3A substrates, which could be important for drugs with a narrow therapeutic window, such as simvastatin, cisapride, ciclosporin, ergotamine alkaloids, fentanyl, pimozide, sirolimus, tacrolimus e quetiapine. Furthermore, it has been demonstrated that the use of potent inducers of CYP3A, such as apalutamide, carbamazepine, enzalutamide, fosphenytoin, lumacaftor, lumacaftor-ivacaftor, mitotane, phenobarbital, phenytoin, primidone, rifampin (rifampicin) and St. John’s wort might substantially decrease olaparib efficacy, reducing its median Cmax of 71% (90% CI, 76%–67%) and the median AUC of 87% (90% CI, 89%–84%); thus the co-administration should be avoided. The efficacy of hormonal contraceptives might be reduced, as olaparib slightly induces CYP1A2 and 2B6 *in vitro*. The liver metabolizes olaparib: after the drug administration, 44% is recovered in urine (of which 15% is unaltered, M15 representing the main metabolite) and 42% in feces (6% unaltered, M12 and M15 being among the most abundant metabolites) (Table 1) (Ang et al., 2010; Dean et al., 2012; Yamamoto et al., 2012; Committee for Medicinal Products for Human, 2014; Mateo et al., 2016; Yonemori et al., 2016; Plummer et al., 2018; Rolfo et al., 2019; Yuan et al., 2019).

## 4 Olaparib in special populations

### 4.1 Renal and liver impairment

In patients with renal impairment, olaparib pharmacokinetic properties are altered, significantly increasing AUC and Cmax. Therefore, a higher exposure might eventually increase toxicity. In clinical studies, no relevant increase in exposure to olaparib was found in case of mild renal impairment. In the NCT01894256 phase I trial, patients received olaparib if they had normal renal function or mild to moderate renal impairment. In patients with moderate reduction of renal function, exposure to olaparib could increase up to 44%; therefore, dose adjustments (e.g., 200 mg twice daily) should be used. In case of severe renal dysfunction, without specific evidence, it is not safe to recommend olaparib (Rolfo et al., 2019).

On the contrary, hepatic dysfunction did not alter olaparib pharmacokinetics, therefore not requiring dose adjustments, except in patients with severe liver impairment, for which no dedicated studies exist; hence, olaparib should not be recommended (Rolfo et al., 2020).

### 4.2 Older patients

Although most OCs develop after age 65, only around 1 out of 3 patients is aged ≥65 in the major clinical trials of olaparib. In an ancillary analysis of ≥65 patients included in olaparib trials, no differences in adverse events (AEs), even those of severe grade, were detected between the older and the younger patients. The discontinuation rate of the two groups stood around 44.7%–64.7% of patients but was not significantly different between the age subgroups (Dockery et al., 2017). We recently performed a meta-analysis, showing no differences in efficacy between older and younger patients, both with single agents and in combination with bevacizumab. Moreover, no increased risk of hematologic toxicity emerged in ≥65 women (Maiorano et al., 2022a). However, only SOLO1, SOLO2, and PAOLA-1 trials published data explicitly focusing on older patients (Moore et al., 2018; Ray-Coquard et al., 2019; Trillsch et al., 2020). Therefore, even if the evidence did not limit the use of full-dose olaparib in the old population, considering the high median age at diagnosis of mOC and the aging population in the next years, trials explicitly focusing on the elder age subgroups should be designed.

## 5 Therapeutic efficacy of olaparib

### 5.1 Advanced BRCA mutant OC after 3 or more lines of chemotherapy

In December 2014, the FDA approved olaparib for treating women with deleterious or suspected deleterious gBRCAm advanced OC who have been previously treated with three or more lines of chemotherapy, based on the results of the phase II trial Study 42 (NCT01078662). The study treated 298 germline BRCA mutant (gBRCAm) cancers, of whom 193 (65%) had OC, with olaparib. They had received at least three lines of CHT, with 39 patients defined as platinum-sensitive (PS), 81 platinum-resistant (PRes), and 14 platinum-refractory (PRef) if the time from completion of last platinum CHT to study start was >6 months, <6 months or <2 months and progressive disease (PD) was the best response to last platinum, respectively. There was no prespecified primary endpoint, but the overall response rate (ORR) and median duration of response (mDoR) were collected first. The overall ORR was 34%. The PS subgroup reached the highest ORR (46%) while in the PRes group, ORR was 30%. The lowest ORR was reached by the PRef subgroup (14%). mPFS was 6.7 months, ranging from 5.5 to 9.4 months in the PRes and the PS groups, respectively (Domchek et al., 2016; Matulonis et al., 2016) (Table 2).

**TABLE 2:** Summary of studies employing Olaparib as maintenance in advanced OC.

Study name (NCT)—year	Phase	Target population (*number of pts*)	Olaparib dosage	Comparative arm	Primary EP	Results
Maintenance in advanced gBRCAm OC after 3 or more lines of chemotherapy						
Study 42 (NCT01078662) - 2010 Domchek et al., (2016); Matulonis et al., (2016)	II	gBRCAm tumors (*n* = 298) 3 or more prior lines of CHT (*n* = 137) PS (*n* = 39) PRes (*n* = 81) PRef (*n* = 14)	400 mg BID	—	ORR mDoR	Overall
						ORR 34% (95% CI, 26%–42%)
						2 CRs (2%)
						44 PRs (32%)
						mDoR 7.9 months (95% CI, 5.6–9.6 months)
						mPFS 6.7 months (95% CI, 5.5–7.6 months)
						PS
						ORR 46% (95% CI, 30%–63%) mDoR 8.2 months (95% CI, 5.6–13.5 months)
						PFS 9.4 months (95% CI, 6.7–11.4 months)
						PRes
						ORR 30% (95% CI, 20%–41%) mDoR 8.0 months (95% CI, 4.8–14.8 months)
						PRef
						ORR 14% (95% CI, 2%–43%) mDoR 6.4 months (95% CI, 5.4–7.4 months)
						PFS 5.5 months (95% CI, 4.2–6.7 months)
Maintenance in PS-ROC						
Study 19 (NCT00753545) - 2012 Ledermann et al., (2012)	II	PS-ROC (*n* = 265)	400 mg BID	PBO	PFS	Overall
		O group (*n* = 136)				PFS 8.4 months vs. 4.8 months (HR 0.35; 95% CI, 0.25–0.49; *p* < 0.001)
		PBO group (*n* = 129)				OS 29.8 months v. 27.8 months (HR 0.88; *p* = 0.44)
		g/sBRCAm (screened *n* = 254)				BRCAm
		O (*n* = 74, 56%)				PFS 11.2 months vs. 4.3 months (HR 0.18; 95% CI, 0.10–0.31; *p* < 0.0001)
		PBO (*n* = 62, 50%)				OS 34.9 months vs. 31.9 months (HR 0.73; *p* = 0.19)
SOLO2/ENGOT-Ov21 (NCT01874353)—2013 Pujade-Lauraine et al., (2017); Poveda et al., (2021)	III	PS-ROC g/sBRCAm (*n* = 294)	300 mg BID	PBO	PFS	PFS 19.1 mos vs. 5.5 months (HR 0.30; 95% CI, 0.22–0.41; *p* < 0.0001)
		O group (*n* = 195)				
		PBO (*n* = 99)				
OPINION (NCT03402841) - 2018 Poveda et al., (2022)	IIIb	PS-ROC gBRCAwt (*n* = 279)	300 mg BID	-	PFS	Overall PFS 9.1 mos tBRCAm PFS 16.4 months
		Biomarker status tBRCAm (*n* = 27)				HRD + including BRCAm PFS 11.1 mos
		tBRCAwt (*n* = 232)				HRD + excluding BRCAm PFS 9.7 months
		HRD+ (*n* = 94)				HRD- PFS 7.3 months
First-line maintenance in newly diagnosed OC						
SOLO1/GOG 3004 (NCT01844986) - 2013 Moore et al., (2018)	III	First-line advanced g/sBRCAm OC after CR or PR to CHT (*n* = 391)	300 mg BID	PBO	PFS	PFS 56 months vs. 13.8 months (HR 0.30; 95% CI, 0.23–0.41; *p* < 0.001)
		O group (*n* = 260)				PFS2 NR vs. 41.9 months (HR 0.50; 95% CI, 0.35–0.72; *p* < 0.001) mOS NR vs. 75.2 months (HR 0.55; 95% CI, 0.40–0.76; *p* = 0.0004)
		PBO group (*n* = 131)				
PAOLA-1/ENGOT-ov25 (NCT02477644) - 2015 Ray-Coquard et al. (2019)	III	First-line advanced OC after CR or PR to CHT (*n* = 806)	300 mg BID plus bevacizumab 15 mg/kg q3w for 15 months	PBO + B	PFS	Overall HR for PFS 0.60 (95% CI, 0.49–0.74)
		O + B (*n* = 537)				HiR group
		PBO + B (*n* = 269)				Overall
		HiR group (74%)				PFS 20.3 months vs. 14.7 months (HR 0.60; 95% CI, 0.49–0.74)
		LoR group (26%)				BRCAm
						PFS US vs. 19.4 months (HR 0.37; 95% CI, 0.23–0.59)
						HRD+ (including BRCAm)
						PFS US vs. 16.0 months (HR 0.39; 95% CI, 0.28–0.54)
						HRD-PFS 15.6 vs. 13.8 months (HR 0.93; 95% CI, 0.68–1.30)
						LoR group
						Overall
						PFS US vs. 22.9 months (HR 0.46; 95% CI, 0.30–0.72)
						BRCAm
						PFS 29.2 months vs. 22.9 months (HR0.11; 95% CI, 0.03–0.31)
						HRD+
						PFS NR vs. 22.1 mos (HR 0.15; 95% CI, 0.07–0.30)

B, bevacizumab; BID, twice a day; BRCA, breast cancer gene; BRCAm, mutated BRCA; BRCAwt, BRCA, wild-type; CHT, chemotherapy; CI, confidence interval; CR, complete response; EP, endpoint; g/s/tBRCAm, germline/somatic/tumor-associated BRCA mutation; HiR, higher risk [subgroup]; HR, hazard ratio; HRD, homologous recombination deficiency [genes]; LoR, lower risk [subgroup]; mos, months; NR, not reached; O, olaparib [arm]; OC, ovarian cancer; OS, overall survival; PBO, placebo [arm]; PFS, progression-free survival; PR, partial response; PRes, platinum resistant; PRef, platinum refractory; PS, platinum sensitive; PS-ROC, platinum sensitive - recurrent ovarian cancer; q3w, once every 3 weeks; US, unstable; vs., *versus*.

### 5.2 Maintenance treatment of recurrent ovarian cancer after complete or partial response to platinum-based chemotherapy

Olaparib is currently indicated for the maintenance treatment of adult patients with recurrent OC in complete or partial response to platinum-based CHT after FDA approval in August 2017 based on Study 19, SOLO2, and OPINION trials (Ledermann et al., 2012; Pujade-Lauraine et al., 2017; LaFargue et al., 2019; Poveda et al., 2021; Poveda et al., 2022).

Study 19 (NCT00753545) was a randomized, phase II study to evaluate maintenance therapy with olaparib in patients with PS-ROC after receiving two or more platinum-based regimens. A pre-planned retrospective analysis of the BRCAm population was later performed and included (Ledermann et al., 2014). The primary endpoint was PFS—by overall population and by BRCA status. 265 patients were enrolled to receive olaparib (*n* = 136) or placebo (PBO—*n* = 129). A significantly longer PFS was observed with olaparib than PBO: mPFS in the overall population was 8.4 *versus* 4.8 months. In the BRCAm population, the benefit of olaparib over PBO was even more remarkable, with mPFS of 11.2 *versus* 4.3 months, if compared with BRCA wild type (BRCAwt) population, reaching an mPFS of 7.4 *versus* 5.5 months. No significant differences in terms of overall survival (OS) emerged. Of note, although the authors did not pre-plan the analysis, efficacy data seemed consistent with the hypothesis that olaparib is effective irrespectively of germline or somatic mutation of BRCA (Domchek et al., 2016; Matulonis et al., 2016).

In the randomized, double-blind, phase III study SOLO2/ENGOT-Ov21 (NCT01874353), evaluating olaparib maintenance in PS-ROC with somatic or germline BRCAm, 294 patients were randomized to olaparib (*n* = 195) or PBO (*n* = 99). The study met its primary endpoint, as PFS was significantly longer in the olaparib subgroup: indeed, mPFS was 19.1 *versus* 5.5 months. The OS data, although immature, showed no detrimental survival for patients receiving olaparib (Pujade-Lauraine et al., 2017; Poveda et al., 2021).

279 patients with gBRCAwt, PS-ROC were enrolled in the phase IIIb OPINION trial (NCT03402841) to receive olaparib. At screening, 264 (94.6%) patients presented gBRCAwt. Retrospective analyses of somatic BRCA mutations also resulted in 37 (13.3%) patients bearing a BRCA mutation, 27 of which had a sBRCAm (9.7%) and 6 (2.2%) with a gBRCAm. Furthermore, among the 232 (83.2%) non-tBRCAm patients - namely, patients not bearing deleterious or suspected deleterious sBRCAm, 94 resulted in HRD (33.7%). 165 (59.1%), 84 (30.1%). PFS was the primary endpoint, while mPFS according to biomarker status (e.g., HRD and tBRCAm), and the number of prior lines of treatment, were secondary endpoints. The overall mPFS was 9.2 months. In the tBRCAm subgroup, mPFS was 16.4 months mPFS was 11.1 months in the HRD group including BRCAm, 9.7 months in the HRD excluding BRCAm, and 7.3 months in the HR proficient (HRP) subgroup. Although the study lacked a PBO comparator group that could quantify the magnitude of olaparib benefit in terms of PFS, it demonstrated the activity of maintenance olaparib in the context of PS-ROC, regardless of HRD or BRCA status (Poveda et al., 2022).

### 5.3 First-line maintenance treatment of either BRCAm or HRD-positive advanced ovarian cancer

Olaparib is also indicated, in combination with bevacizumab, for the maintenance treatment of women with advanced OC after CR or PR to first-line platinum-based CHT, bearing HRD and/or BRCA mutation (Arora et al., 2021). FDA approved in December 2018, based on the pivotal results of the randomized, phase III clinical trial SOLO1/GOG 3004, employing olaparib (*n* = 260) *versus* PBO (*n* = 131). The primary endpoint was PFS, while the second-interval PFS (PFS2) and OS were secondary endpoints. 5-year PFS rate was 60% in the olaparib and 27% in the PBO group, mPFS was 56 months in the olaparib *versus* 13.8 months in the PBO group. PFS2 rate was 75% in the olaparib and 60% in the PBO group, and mPFS2 was NR in the olaparib and 41.9 months in the PBO group. The OS analysis was recently updated after a 7-year follow-up, showing that 67.0% of patients in the olaparib group were still alive compared with 46.5% in the PBO group. (Moore et al., 2018; DiSilvestro et al., 2023).

Furthermore, in the phase III PAOLA-1/ENGOT-ov25 trial (NCT02477644), 806 patients with advanced newly diagnosed advanced OC, with CR or PR to platinum-based CHT, were randomized to receive olaparib plus bevacizumab (*n* = 537) or PBO plus bevacizumab (*n* = 269). In this analysis, patients were divided into a higher-risk subgroup (HiR—74%) in case of surgery performed on a FIGO stage III disease with residual disease or neoadjuvant chemotherapy administered or FIGO stage IV disease, and a lower-risk subgroup (LoR—26%), with radical surgery performed on a FIGO stage III disease. BRCA status was assessed only on tumor samples; thus, germline BRCA status was unknown. After a median follow-up of 22.9 months, PFS favored the olaparib plus bevacizumab group in both risk subgroups, thus confirming the benefit of olaparib as in SOLO1, and showing, in addition, the efficacy of the combination with bevacizumab. In fact, based on the PAOLA-1 results, the combination was approved by FDA in May 2020. In the HiR subgroup, mPFS was 20.3 *versus* 14.7 months. In the LoR subgroup, HR for PFS was 0.46 in the olaparib plus bevacizumab group. At the same time, the mPFS was inestimable in the olaparib plus bevacizumab group *versus* 22.9 months in the PBO group. Among the HiR BRCAm patients, mPFS was inestimable for the olaparib plus bevacizumab group *versus* 19.4 months in the PBO group, while in the lower-risk mBRCA patients, mPFS was 29.2 *versus* 22.9 months. In HRD patients mPFS was not estimable *versus* 16.0 months in the HiR subgroup, while in the LoR subgroup, mPFS was NR vs 22.1 months. Considering the HiR HRP patients, mPFS was 15.6 *versus* 13.8 months. No benefit in terms of PFS among LoR HRP patients derived from olaparib plus bevacizumab. PAOLA‐1 was more representative of advanced OC patients than SOLO1, as patients’ selection was not based on BRCA status. The PFS benefit observed with olaparib plus bevacizumab in patients with tBRCAm tumors in the PAOLA‐1 appears consistent with the SOLO‐1 results, supporting the efficacy of olaparib in BRCAm tumors regardless of somatic or germline mutation origin (Ray-Coquard et al., 2019; González-Martín et al., 2022; Harter et al., 2022).

## 6 Tolerability of olaparib

Hematological toxicities are common class effects of PARPis, representing the most common cause of dose modification, interruption, and discontinuation. They tend to occur early after treatment start and to recover after a few months. Anemia, usually the most common among haematologic AEs, might be related to PARP2 inhibition that affects the differentiation of erythroid progenitors, reducing erythrocytes’ life expectancy in mice, even if erythropoietin plasma concentrations are increased, thus suggesting that supplementation might not be the best therapeutic option to manage anemia in these patients. On the contrary, transfusions are generally recommended for symptomatic anemia and hemoglobin values less than 7 g/dL. A baseline blood count should be obtained before starting olaparib and monitored monthly, at least during the first year of treatment. Olaparib should not be restarted if hematologic toxicity results > G1 (e.g., haemoglobin<10 g/dL, neutrophils <1,500/mm^3^, platelets <75,000/mm^3^) from previous therapy (Committee for Medicinal Products for Human, 2014; US Food and Drug Administration FDA, 2014; EMA Olaparib product information, 2023). A bone marrow analysis is recommended if severe hematologic toxicity lasts over 4 months. As the fundamental mechanism of PARP inhibition is interfering with DNA repair pathways, another severe class effect, although rare, is the onset of secondary malignancies, namely, myelodysplastic syndrome (MDS) and acute myeloid leukemia (AML), with an incidence of 0.5%–1.4%, usually after long-term treatment. The true incidence of SPMs after PARPis is difficult to estimate, as almost all patients also received other DNA-damaging drugs, such as platinum-based CHT (LaFargue et al., 2019). The risk of developing new second primary malignancies (SPMs), reported in 0.7%–2% of patients in the SOLO2, OPINION, SOLO1, and PAOLA-1—especially breast, thyroid, and rectal cancers, was not found to be increased in the olaparib group in a recent meta-analysis of 23 randomized clinical trials, thus suggesting no additional close monitoring of patients treated with PARPis. Among 8,857 patients included in the analysis, 51 SPMs were reported in the PARPis (0.9%) and 24 in the PBO group (0.7%). PARPis exposure was not associated with an increased risk of developing SPM *versus* PBO (*p* = 0.62) after up to 78 months of follow-up (Pujade-Lauraine et al., 2017; Moore et al., 2018; Ray-Coquard et al., 2019; Morice et al., 2021; Poveda et al., 2021; Poveda et al., 2022).

Gastrointestinal toxicities are also very commonly associated with PARPis, and patients should be aware of the high incidence of nausea to prevent its occurrence prophylactically. To lessen symptoms, daily prokinetic and antihistamine drugs can be administered. Persistent nausea or vomiting can be managed using various antiemetic drugs, such as metoclopramide, prochlorperazine, phenothiazine, dexamethasone, olanzapine, haloperidol, or lorazepam. The neurokinin-1 receptor antagonist, aprepitant, should be avoided with olaparib since it strongly inhibits CYP3A4, thus affecting olaparib plasma concentrations. Fatigue and asthenia also seem to be a class effect and can be managed using non-pharmacological approaches, such as exercise, massage therapy, and cognitive and behavioral therapy. The use of psychostimulants such as methylphenidate and ginseng is currently being investigated. Of note, it is confirmed by several animal studies that olaparib is embryo-toxic and teratogenic and, thus, should be avoided during pregnancy. In addition, fertile women should avoid pregnancy during treatment and at least 6 months after olaparib stops and thus be counseled about birth control. Breastfeeding is also contraindicated during treatment and until 2–4 weeks after the last dose of olaparib (LaFargue et al., 2019). Analyzing the tolerability of olaparib as maintenance therapy in advanced OC, we found a median duration of treatment ranging from 5.6 to 22.6 months, while if considering the PBO arms, from 5.6 to 19.8 months. Almost every patient experienced any grade AEs, ranging from 95.6% to 99% of patients receiving olaparib and from 90.6% to 96% of patients in the PBO arms. Focusing on the olaparib arms, nausea was the most commonly reported all-grade AEs, ranging from 60% to 75.9%, followed by fatigue/asthenia (48.5%–64%), vomiting (22%–44%), diarrhea (14.3%–35%) while, among the haematologic toxicity, anemia was by far the most commonly reported, ranging from 16.9% to 43.6%. However, if considering only ≥ G3 AEs, reported by 29%–57% of patients treated with olaparib *versus* 19%–51% of patients receiving PBO, hematological toxicities were the most frequent, with ≥G3 anemia as the most common by far, ranging from 5.1% to 22%. Neutropenia ranged from 0% to 9%, and thrombocytopenia from 1% to 2.2%. ≥G3 fatigue ranged from 3.2% to 7.3%, and abdominal pain from 0% to 8%, while nausea, vomiting, and diarrhea were experienced only by less than 5% of patients. Anaemia was the most frequent AE that led to treatment discontinuation, which occurred in 2.2%–25% of patients receiving olaparib *versus* 0.7%–6% of the PBO group. AEs were managed with dose interruptions (27.9%–60% *versus* 8.6%–26%) or reductions (22%–41% *versus* 3%–7%) rather than discontinuation.

Considering the safety data from olaparib studies, we found that, in Study 42, the median treatment duration was 168 days 43% of dose interruptions were reported, 22% of dose reductions and 5% of patients discontinued treatment. 98% of patients experienced AEs of any grade, while 55% experienced ≥ G3 AEs. The most common any-grade AEs were nausea (60%), fatigue (55%), vomiting (44%), anemia (34%), abdominal pain (29%), and diarrhea (30%), while the most common ≥ G3 AEs were anemia (20%), abdominal pain (8%), fatigue (7%) and dyspnea (4%) (Domchek et al., 2016; Matulonis et al., 2016). In Study 19, the median treatment duration was 206.5 days with olaparib and 141 days with PBO. 95.6% and 90.6% of patients developed any-grade AEs in the olaparib and PBO groups, respectively. Among patients in the olaparib group, the most common AEs were nausea (68.4%), fatigue (48.5%), vomiting (31.6%), diarrhea (22.8%), abdominal pain (17.6%), anemia (16.9%). ≥G3 AEs occurred in 35.3% of patients treated with olaparib *versus* 20.3% of patients receiving PBO, most commonly fatigue (6.6%), anemia (5.1%), nausea/vomiting/diarrhea (each 2.2%), and abdominal pain (1.5%). In the olaparib group, 27.9% and 22.8% of patients experienced dose interruption or reductions (vs 8.6% and 4.7% of the PBO group). Three patients in the olaparib group permanently discontinued treatment *versus* one treatment interruption with PBO. No deaths were recorded (Ledermann et al., 2012). In the SOLO2/ENGOT-Ov21 trial, the median treatment duration was 19.4 months with olaparib and 5.6 months with PBO. 98.5% of patients in the olaparib group and 94.9% in the PBO group experienced any grades AEs, with 36.9% and 18.2% experiencing ≥ G3 AEs, respectively. The most common all-grade toxicities were nausea (75.9% vs 33.3%), fatigue/asthenia (65.6% vs 39.4%), anemia (43.6% vs 8.1%), vomiting (37.4% vs 19.2%), and diarrhea (32.8% vs 20.2%). However, anemia was the most common ≥ G3 AE (19.5% vs 2.0%), while the incidences of ≥G3 neutropenia (5.1% vs 4.0%) and thrombocytopenia (both 1.0%) were not significantly increased in the olaparib subgroup. SOLO2 had a higher incidence of anemia than Study 19, which could be explained by more prolonged exposure to olaparib for patients in this study. Of note, one patient (0.5%) of the olaparib group experienced AML, resulting in death. The long-term incidence of AML, MDS, and chronic myelomonocytic leukemia (CMML) was 2.1% with olaparib and 4.0% with PBO. 45.1% and 18.2% of patients in the olaparib and PBO groups required dose interruptions, while 25.1% and 3.0% required dose reductions due to AEs, respectively. 10.8% of patients in the olaparib and 2.0% in the PBO group discontinued treatment because of toxicity, mainly anemia (3.1%) and neutropenia (1.0%) (Pujade-Lauraine et al., 2017; Poveda et al., 2021).

**FIGURE 1:**
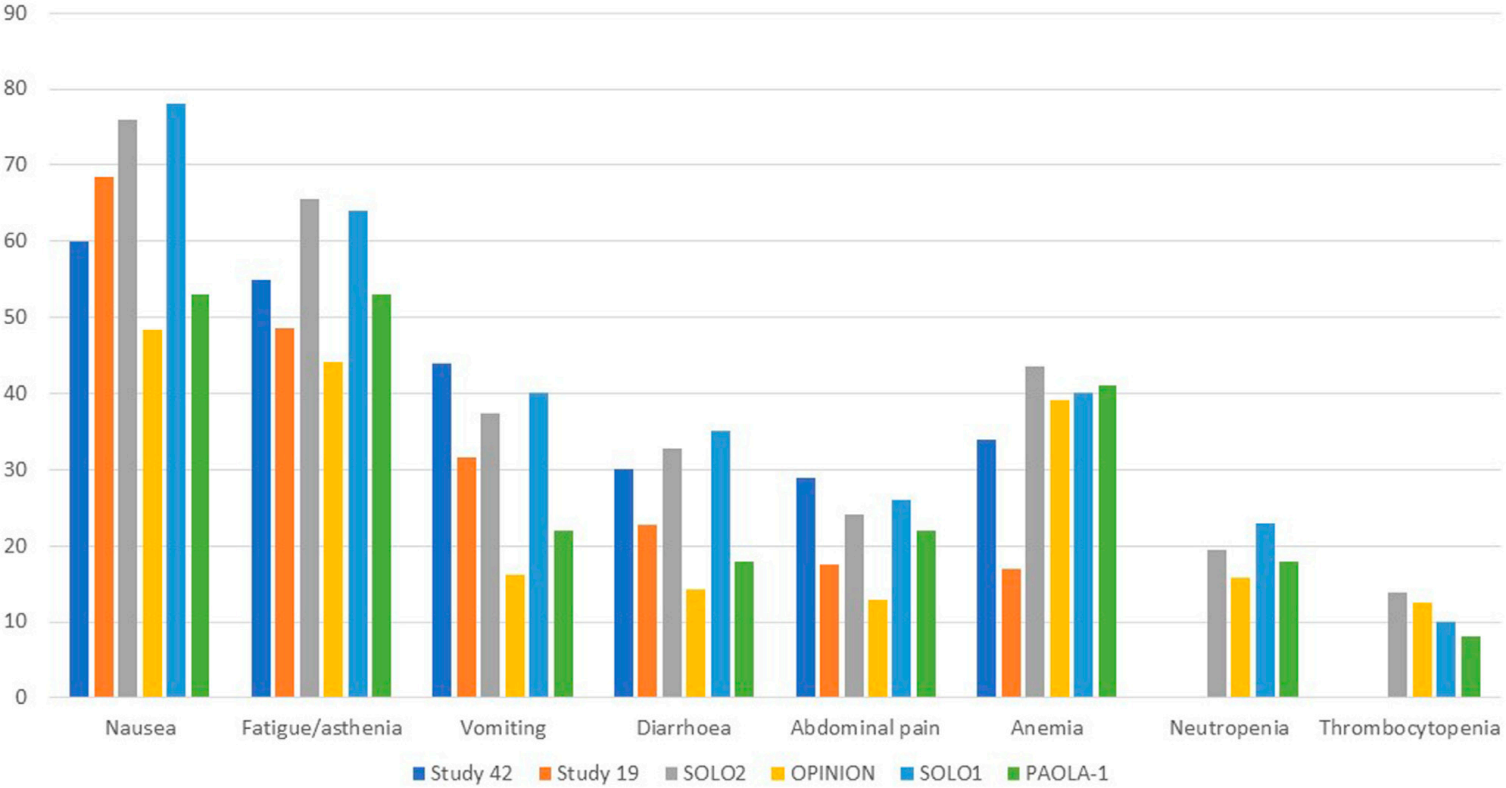
Most frequent all-grades adverse events during olaparib therapy.

**FIGURE 2:**
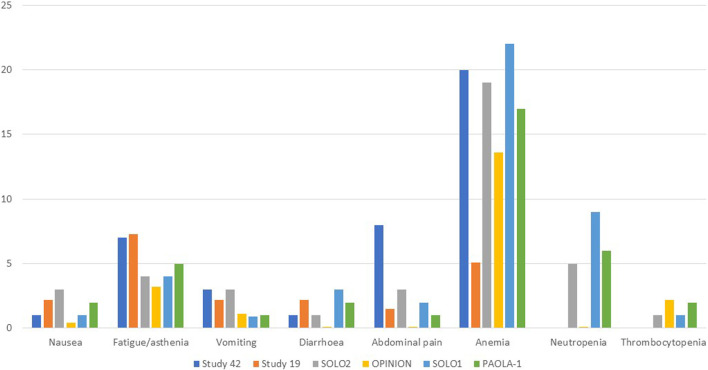
Most frequent ≥G3 adverse events during olaparib therapy.

All grades and ≥G3 AEs were reported in 95.7% and 29.0% of patients in the OPINION trial, respectively. Nausea (48.4%), fatigue/asthenia (44.1%), anemia (39.1%), and diarrhea (14.3%) were the most common AEs of all grades, while anemia (13.6%) and fatigue/asthenia (3.2%) were the most common ≥ G3 AEs. Dose interruption, dose reduction, and treatment discontinuation were applied to 47.0%, 22.6%, and 7.5% of patients. The median treatment duration was 9.4 months. Anaemia (1.8%), decreased platelet count, depression, fatigue/asthenia, and thrombocytopenia (0.7% each) were the most common AEs leading to treatment discontinuation. MDS and SPMs (mainly rectal and breast cancer) were reported in 0.7% of patients each (Poveda et al., 2022). 98% of olaparib and 92% of PBO patients of the SOLO1 trial experienced AEs of any grade, among which ≥ G3 AEs were reported in 40% and 19% of patients. Nausea (78% and 38%), fatigue/asthenia (64% and 42%), vomiting (40% and 15%), anemia (40% and 10%), and diarrhea (35%) were the most common all-grade AEs. The most frequent ≥ G3 AE was anemia, which occurred in 22% of olaparib and 2% of PBO patients. Dose interruptions occurred in 52% of olaparib vs 17% of PBO patients, while dose reductions occurred in 29% vs 3%. Discontinuations were less frequent with olaparib (12%) than with PBO (3%). One (1%) fatal AML occurred over 30 days after olaparib discontinuation. Of note, 2% of olaparib patients developed SPMs (breast, oral cavity, and thyroid), and 2% of PBO patients developed SPMs (breast cancer) (Moore et al., 2018). Finally, in the PAOLA-1 trial, the median duration of treatment was 16.6 months for olaparib plus bevacizumab and 13.4 months for PBO in the HiR group, while for the LoR group, 22.6 vs19.8 months 99% and 96% of patients experienced AEs, with olaparib plus bevacizumab and PBO plus bevacizumab, respectively. 57% of patients experienced severe AEs with olaparib plus bevacizumab vs 51% in the PBO/bevacizumab arm, showing no significant safety differences among all subgroups. Fatigue or asthenia (53% vs 22%), nausea (53% vs 22%), hypertension (46% vs 60%), and anemia (41% vs 10%) were the most frequent all-grade AEs. Hypertension (19% vs 30%) and anemia (17% vs 1%) were the most frequently reported ≥ G3 AEs. Dose interruptions occurred in 53% vs 26% of HiR patients and 60% vs 21% of LoR patients, while discontinuation in 19% vs 6% in the HiR and 25% vs 5% in the LoR subgroups. One patient (0.3%) receiving olaparib/bevacizumab and 2 (1%) receiving PBO/bevacizumab experienced fatal AEs. A total of 6 patients (1%) in the olaparib/bevacizumab and 1 (<1%) in the PBO/bevacizumab group developed AML or MDS, while 7 patients (1%) and 3 (<1%) developed SPMs (Ray-Coquard et al., 2019). Figures 1, 2 resume the most frequent all-grades and ≥G3 AEs during olaparib treatment. Table 3 enlists the main AEs grouped according to CTCAE (Common Terminology Criteria for Adverse Events) grading.

**TABLE 3:** Adverse events of Olaparib in clinical trials according to CTCAE.

	Study 42		Study 19		SOLO2		OPINION		SOLO1		PAOLA-1	
AEs	All grades (*%*)	≥G3 (*%*)	All grades (*%*)	≥G3 (*%*)	All grades (*%*)	≥G3 (*%*)	All grades (*%*)	≥G3 (*%*)	All grades (*%*)	≥G3 (*%*)	All grades (*%*)	≥G3 (*%*)
Nausea	60	1	68.4	2.2	75.9	3	48.4	0.4	78	1	53	2
Fatigue	55	7	48.5	6.6	65.6	4	44.1	3.2	64	4	53	5
Vomiting	44	3	31.6	2.2	37.4	3	16.1	1.1	40	<1	22	1
Diarrhoea	30	1	22.8	2.2	32.8	1	14.3	14.3	35	3	18	2
Abdominal pain	29	8	17.6	1.5	23	3	12.9	12.9	25	2	19	1
Anemia	34	20	16.9	5.1	43.6	19	39.1	13.6	40	22	41	17
Neutropenia	NA	NA	NA	NA	19	5	15.8	1.8	11	9	18	6
TCP	NA	NA	NA	NA	14	1	12.5	2.2	11	1	8	2

AE(s), adverse event(s); G3, grade 3; NA, not available; TCP, thrombocytopenia.

## 7 Future perspectives and conclusions

PARPis have transformed the therapeutic landscape of advanced OC in the last decade, and olaparib was a pioneer drug in this field. We provided an overview of the clinical and pre-clinical characteristics of olaparib, synthesizing the results of trials that led to its approval in different settings and analyzing its safety profile. Olaparib resulted in effective maintenance therapy in the recurrent and newly diagnosed advanced OC setting in all patients’ subgroups, regardless of BRCA status, with a generally good safety profile and quality of life. Some queries, however, remain unanswered and are currently being investigated by new ongoing trials, mainly the combination with different agents, and the use of olaparib in the platinum-resistant setting.

Combination studies are trying to meet the need for new therapeutic approaches, increasing the potential for new or augmented adverse events. An exciting strategy, currently under investigation, is to combine PARPis with immune checkpoint inhibitors (ICIs), with a strong rationale behind this combination. In fact, PARPis upregulate Programmed death-ligand 1 (PD-L1) expression; they interact with the tumor microenvironment, being able to switch it towards an immune-responsive state and increase tumor-infiltrating lymphocytes. Moreover, through DNA damage, PARPis stimulate neo-antigen production, therefore augmenting the tumor mutational burden. PARPis also switch on the STING pathway that, on its hand, reinforces interferon-γ dependent immune cells (Maiorano et al., 2022b). The combination of olaparib and the anti-PD-L1 durvalumab was tested in two ongoing phase II trials, reporting strong response rates. In the context of PS-ROC BRCAm OC, the MEDIOLA study reported an ORR of 71.9%, mOS NR, and mPFS of 11.1 months (Drew et al., 2019). Subsequently, the study randomized 63 BRCAwt patients to durvalumab plus olaparib with or without bevacizumab. The doublet cohort reached an ORR of 31.3%, and the triplet cohort of 77.4% (Drew et al., 2020). A final mOS analysis presented at ESMO2022 showed an mOS of 23.3 months vs 31.9 months in the doublet and triplet cohorts, respectively (Banerjee et al., 2022). The same combination was administered in the NCT02484404 phase II trial, with an ORR of 14% and an mPFS of 3.0 months (Lampert et al., 2020). The NCT02571725 phase Ib/II trial investigated the combination of olaparib with the anti-Cytotoxic T-lymphocyte-associated protein 4 (CTLA4) tremelimumab. Only 3 patients were treated, all of them achieving a PR (Adams et al., 2017) (Table 4).

**TABLE 4:** Results of studies employing olaparib and ICIs.

Study name (NCT)	Phase	Target population (*number of patients*)	Combination	Results
NCT02484404 Lampert et al., (2020)	II	ROC (*n* = 35: 30 PR*-*ROC +5 PS-ROC)	Olaparib plus durvalumab (anti-PD-L1)	ORR 14% mPFS 3.0 months
		BRCAwt (*n* = 27) gBRCAmut (*n* = 6)		
		sBRCAmut (*n* = 2)		
MEDIOLA Banerjee et al., (2022)	II	PS-ROC gBRCAmut (*n* = 32)	Olaparib plus durvalumab	ORR 71.9% mPFS 11.1 mos
				mOS NR
		PS-ROC BRCAwt	Olaparib plus durvalumab	ORR 31.3% mPFS 5.5 months
				mOS 23.3 months
		PS-ROC BRCAwt	Olaparib plus bevacizumab plus durvalumab	ORR 77.4% mPFS 14.7 months
				mOS 31.9 months
NCT02571725 Adams et al. (2017)	Ib/II	gBRCAmut ROC (*n* = 3)	Olaparib plus tremelimumab (anti-CTLA4)	ORR 100%

BRCA, breast cancer associated gene; BRCAwt, BRCA, wild-type; CTLA4, cytotoxic T.lymphocyte-associated protein 4; gBRCAmut, germline mutated BRCA; mos, months; NR, not reached; ORR, overall response rate; PD-L1, programmed death-ligand 1; PFS, progression free survival; PR/PS-ROC, platinum-resistant/platinum-sensitive recurrent ovarian cancer; ROC, recurrent ovarian cancer; sBRCAmut, somatic mutated BRCA.

The rationale behind the combination of PARPis and anti-angiogenic drugs stands on two main mechanisms: PARP inhibition decreases angiogenesis; hypoxia and Vascular endothelial growth factor receptor 3 (VEGFR3) inhibition also induce the downregulation of HR proteins (Bindra et al., 2005; Tentori et al., 2007; Lim et al., 2014). PAOLA-1 already showed the efficacy and safety of the combination of olaparib and bevacizumab (Ray-Coquard et al., 2019). A phase II trial combining cediranib with olaparib *versus* olaparib alone in PS-ROC showed a significantly better mPFS in the combination group (17.7 vs 9.0 months) (Liu et al., 2014). NRG-GY004, a phase III randomized clinical trial, compared the efficacy of olaparib, with or without cediranib, *versus* platinum-based CHT in PS-ROC. However, in this study, olaparib/cediranib did not improve PFS *versus* chemotherapy regardless of BRCA status, but increased AEs (Liu et al., 2022).

OC with a “BRCAness” phenotype exhibits a higher sensitivity to both platinum and PARPis, than OC without a “BRCAness” phenotype. Hence, platinum sensitivity might represent a potential biomarker for olaparib sensitivity. In fact, the clinical benefit rate of olaparib fell from 69.2% in platinum-sensitive to 45.8% in platinum-resistant and 23.1% in platinum-refractory BRCA1/2-mutated OC (Fong et al., 2010). In BRCA1/2 wild-type OC, half of the platinum-sensitive patients responded to olaparib *versus* only 4% of the platinum-resistant women. However, a response to platinum does not always guarantee a response to olaparib. Indeed, differently from PARPis, platinum sensitivity results from defective nucleotide excision repair (NER) (Ceccaldi et al., 2015). The platinum-induced DNA cross-links are highly deleterious and more cytotoxic than the SSBs caused by PARPis. In addition, the partial restoration of HR is insufficient to repair the cross-links caused by platinum salts. Therefore, such OCs retain platinum sensitivity but exhibit PARPis resistance (Lord and Ashworth, 2013). It has also been evidenced that an increased platinum-to-platinum interval during olaparib treatment is associated with a response to subsequent platinum treatment [ (Ang et al., 2013), (Norquist et al., 2011). As for the platinum-resistant recurrent OC (PR-ROC) setting, patients relapsing within 12 months of platinum-based CHT usually have a poorer response to subsequent treatments (Markman et al., 2004). Several trials involving PR-ROC patients have not yet resulted in improved responses or benefits in terms of survival, thus justifying further experimental work and clinical trials with novel agents. The phase II BAROCCO trial (NCT03314740) compared weekly paclitaxel with the olaparib-cediranib combination in PR-ROC, not significantly impacting PFS) (Colombo et al., 2022). Clinical activity of the olaparib-cediranib combination was shown by the phase IIb CONCERTO trial, with 60 BRCAwt PR-ROC reaching an ORR of 15.3%, an mPFS of 5.1 months, and a mOS of 13.2 months (Lee et al., 2022). The same combination is also being investigated in the phase II OCTOVA trial (NCT03117933) (Mansouri et al., 2021). The GEICO1601-ROLANDO phase II trial (NCT03161132) will assess the efficacy of olaparib with pegylated liposomal doxorubicin (PLD) in PR-ROC, regardless of BRCA status, while the randomized phase II CLIO/BGOG-ov10 trial compared olaparib monotherapy vs physicians’ CHT of choice (PLD, Topotecan, Paclitaxel or Gemcitabine) in 100 PR-ROC patients. Olaparib monotherapy showed higher efficacy than CHT in the PR-ROC setting, with an ORR of 17.9% vs 6.1% for olaparib *versus* CHT. Even in heavily pretreated PR-ROC, ORR was 22.9% for olaparib *versus* 0% for CHT. mPFS in PR-ROC was not significantly improved (Perez-Fidalgo et al., 2019; Vanderstichele et al., 2022).

PARP1 has currently been identified as a more significant driver of synthetic lethality than PARP2 (Murai et al., 2012). Therefore, a new generation of highly-selective PARP1-inhibitors is under development. AZD5305 is a first-in-class PARP1-inhibitor and trapper (Johannes et al., 2021; Illuzzi et al., 2022; Zheng et al., 2023). Preliminary results of the phase I/IIa PETRA study (NCT04644068) in patients with BRCA1/2, PALB2, RAD51C/D mutations have been recently presented. Around half of 61 patients with OC (*n* = 19) had PR or SD to AZD5305. The drug’s safety profile is of particular interest, as no discontinuations occurred. The most common AEs were nausea (34%), anemia (21.3%), neutropenia, and TCP (18%). 14.8% of patients experienced ≥ G3 AEs (Yap et al., 2022). This is in line with mouse models, in which the PARP1 selectivity was associated with a more manageable safety profile than common PARPis (Johannes et al., 2021; Illuzzi et al., 2022; Zheng et al., 2023).

In summary, olaparib displays clinical activity and is therefore approved as maintenance treatment of OC starting from the first line, as monotherapy in BRCA mutant, and combined with bevacizumab in HRD patients, and in the PS-ROC independent from BRCA status, with a good balance between efficacy and safety. Further studies are required to expand this drug’s therapeutic application and better select patients most likely to benefit from olaparib.
